# New Hope in Brain Glioma Surgery: The Role of Intraoperative Ultrasound. A Review

**DOI:** 10.3390/brainsci8110202

**Published:** 2018-11-19

**Authors:** Maria Angela Pino, Alessia Imperato, Irene Musca, Rosario Maugeri, Giuseppe Roberto Giammalva, Gabriele Costantino, Francesca Graziano, Francesco Meli, Natale Francaviglia, Domenico Gerardo Iacopino, Alessandro Villa

**Affiliations:** 1 Department of Experimental Biomedicine and Clinical Neurosciences, School of Medicine, Neurosurgical Clinic, AOUP “Paolo Giaccone”, 90100 Palermo, Italy; mariangelapino@live.it (M.A.P.); robertogiammalva@live.it (G.R.G.); francesca.graziano03@unipa.it (F.G.); gerardo.iacopino@gmail.com (D.G.I.); 2 Division of Neurosurgery, IRCCS Neuromed, 86077 Pozzilli, Italy; alessia.imperato@gmail.com; 3 Division of Neurosurgery, ARNAS Civico Hospital, 90100 Palermo, Italy; irene.musca93@gmail.com (I.M.); gabcostantino@gmail.com (G.C.); melifra75@gmail.com (F.M.); francaviglianatale@gmail.com (N.F.); alessandrovilla83@gmail.com (A.V.)

**Keywords:** intraoperative ultrasound, IOUS, brain tumor, glioma surgery

## Abstract

Maximal safe resection represents the gold standard for surgery of malignant brain tumors. As regards gross-total resection, accurate localization and precise delineation of the tumor margins are required. Intraoperative diagnostic imaging (Intra-Operative Magnetic Resonance-IOMR, Intra-Operative Computed Tomography-IOCT, Intra-Operative Ultrasound-IOUS) and dyes (fluorescence) have become relevant in brain tumor surgery, allowing for a more radical and safer tumor resection. IOUS guidance for brain tumor surgery is accurate in distinguishing tumor from normal parenchyma, and it allows a real-time intraoperative visualization. We aim to evaluate the role of IOUS in gliomas surgery and to outline specific strategies to maximize its efficacy. We performed a literature research through the Pubmed database by selecting each article which was focused on the use of IOUS in brain tumor surgery, and in particular in glioma surgery, published in the last 15 years (from 2003 to 2018). We selected 39 papers concerning the use of IOUS in brain tumor surgery, including gliomas. IOUS exerts a notable attraction due to its low cost, minimal interruption of the operational flow, and lack of radiation exposure. Our literature review shows that increasing the use of ultrasound in brain tumors allows more radical resections, thus giving rise to increases in survival.

## 1. Introduction

Maximal safe resection represents the gold standard for surgery of malignant brain tumors: gross total resection of the tumor while preserving the surrounding functional brain tissue is the main goal, since it is associated with longer survival and better patient quality of life [1]. This is particularly true for gliomas, the most common primary malignant brain tumors whose pathogenesis is still unknown [2,3].

Concerning gross total resection, accurate localization and precise delineation of the tumor margins are required in order to avoid devastating lesions on nervous structures [4]. Frame-based and frameless stereotactic preoperative data-based techniques, also known as neuronavigation systems, are routinely used to help surgeons plan the site of craniotomy and identify critical neural structures. Unfortunately, these systems have inherent problems related to loss of accuracy resulting from unpredictable distortions, shifts, and deformations after craniotomy and tissue removal. Therefore, neuronavigation is not a true real-time system: the accuracy is maximal before the craniotomy and decreases significantly while progressing through surgical manipulation. It is due to two main factors: the first is “brain shift” caused by the effect of gravity on the brain, brain swelling, as well as the drainage of cerebrospinal fluid (CSF); the second factor is the deformation of brain parenchyma caused by surgical maneuvers and tumor removal [5].

Several surgical techniques and technological innovations have been recently introduced to help the surgeon achieve the maximal safe resection of the tumor while reducing the odds of post-operative complications [6,7]. Some examples are represented by the intraoperative use of neurophysiological monitoring and the use of the awake surgery technique. Moreover, in recent years, new methods, including intraoperative diagnostic imaging (Intra-Operative Magnetic Resonance-IOMR, Intra-Operative Computed Tomography-IOCT, Intra-Operative Ultrasound-IOUS) and dyes (fluorescence) have become relevant in neuro-oncological surgery, allowing for a more radical and safer tumor resection [8].

Some of these innovations, such as the IOMR, are expensive and not available in every neurosurgical centre. Therefore, in the last few years, great attention has been paid to the possible intraoperative use of ultrasound (IOUS) guidance for brain tumor surgery. IOUS is not a new technology, but it is accurate in distinguishing tumor from normal parenchyma, and allows a real-time intraoperative visualization. IOUS is cheap, easily repeatable, safe for the patient, potentially available in all neurosurgical centres. 

As early as in the 1970s, B-mode ultrasound (US) was introduced into the operating room. However, it was the development and application of real-time grey-scale US imaging technology that really provided an impetus to IOUS. The use of ultrasound to achieve maximally safe resection of brain tumors has been implemented since the 1980s [9]. In the pre-MR era, US (2-dimensional (2D) B-mode grey-scale US) imaging became a routinely-used adjunct in the neurosurgical operating room. With the introduction of MR imaging (MRI), an entirely new and vivid “image” of intracranial anatomy was unveiled. Neurosurgeons rapidly adopted MRI into routine clinical diagnostic practice. The development of stereotactic localization and navigation technology in the late 1980s ushered in the era of “computer-assisted surgery.” In 1992, Le Roux et al. noted that the majority of brain tumors, including low-grade gliomas, were visible with intraoperative ultrasound [10]. The Norwegian Group first employed IOUS in 1997, and demonstrated the utility of real-time intraoperative ultrasound to identify tumors and facilitate resection [11]. In the contemporary era, IOUS imaging is used in neuro-oncological surgery to investigate the spatiotemporal change of the tumor morphology during the operation. B-mode is the most commonly-used modality [12]. The solid part of the tumor appears hyperechogenic compared to the surrounding cerebral parenchyma, while cysts appear hypoechoic. The presence of blood or calcifications, which can often occur in the tumor mass, alters the echogenicity of the lesions; therefore, the procedure should be led by an experienced operator. Many authors described the ultrasonographic characteristics of some tumors by comparing them with conventional imaging studies, such as Computed Tomography (CT) and MR [13,14,15,16,17]. 

After the craniotomy is performed, the utility of ultrasound is evident in different phases of surgery. IOUS help to plan the opening of the dura and to centre the corticectomy. In the following phases, IOUS allows identification of the tumor margins, thus helping for a maximally radical resection. 

The main advantage of IOUS consists of obtaining a real-time scan: this can be repeated as many times as necessary, in order to overcome the errors produced by brain shift [18]. It also avoids the cost and the duration of other intraoperative techniques. 

Its main limitations are spatial resolution, width and orientation of the field of view (different from the standard orthogonal planes of CT and MRI), and scan quality, which are operator dependent. Even if US are widely adopted to evaluate cerebral blood flow through transcranial Doppler [19], Neurosurgeons are not confident with a technique that provides brain images oriented on infinite axis (not only the standard axial, sagittal, and coronal plane), and the ability to “understand and interpret” anatomic details displayed on the screen requires significant training and experience. Since most neurosurgeons do not receive specific US training, and US is not yet a standard diagnostic and intraoperative tool for cerebral lesions, there is an inherent difficulty in interpreting IOUS imaging and in correctly setting up the machine; these two factors both lead to a longer learning curve. Real-time intraoperative fusion of preoperative MRI/CT scans and intraoperative ultrasounds is a highly desirable solution to overcome the above-mentioned limitations.

Moreover, the greatest limitation of US is their limited ability to penetrate the skull; therefore, the most important application of IOUS in brain tumor surgery occurs after performing the craniotomy.

Since IOUS has gained great popularity in recent years, the purpose of this study is to review the current literature to evaluate the role of IOUS in gliomas surgery and to outline specific strategies to maximize its efficacy.

## 2. Literature Research and Findings

We performed a literature research through the Pubmed database by selecting each article focused on the use of IOUS in brain tumor surgery, and in particular, in glioma surgery, published in the last 15 years (from 2003 to 2018). 

We performed a query using the following combinations of the Medical Subject Headings (MESH) terms: ultrasound, intraoperative ultrasound, intraoperative image guidance, glioma surgery, brain tumors surgery, high-grade glioma surgery, low-grade glioma surgery, alternating all these terms in various combinations. 

We included all the studies with information on the diagnosis, the extent of the resection, and the postoperative evaluation of the degree of resection by neuroimaging. 

We excluded all papers written in languages other than English and all studies with incomplete data.

After the identification of all articles that met the inclusion criteria, and after removing duplicates, we selected 39 papers dealing with the use of IOUS in brain tumor surgery, including gliomas.

Twenty-one studies were prospective and 18 were retrospectives. In particular, we focused on the usefulness of ultrasound in the surgery of cerebral gliomas. 

2264 patients were included in our review: 1650 patients with a histologic diagnosis of brain glioma and 522 patients with a non-glial tumor. We did not find any information about the histological report of 94 patients [20]. 

We paid special attention to the evaluation of the postoperative extent of resection (EOR), and in particular, to the achievement of gross-total resection (GTR), defined as the absence of any residual enhancement on postoperative volumetric-enhanced MRI performed within 72 h after surgical resection. 

Another important point was the analysis we performed of the usefulness of IOUS used with or without other neuroimaging techniques. The results of our literature review are briefly summarized in Table 1 (see Section 3).

## 3. The Use of IOUS in Brain Glioma Surgery

The use of IOUS seems to have significantly increased the GTR rate achieved in brain tumors/gliomas surgery.

As regard intraoperative visualisation of the tumor and its residuals, the effectiveness of IOUS has been documented in a series of 192 HGG patients, in which the combination of neuronavigation and IOUS was also related to increased overall survival [21]. Erdogan et al. [22], in a prospective study of 32 patients, documented a good level of agreement between intraoperative ultrasonography and postoperative contrast-enhanced MRI in detecting tumor residue; they concluded that the IOUS produces results similar to those of MRI, and therefore, can be used to maximize tumor resection. 

Regarding the reliability of intraoperative ultrasound images, the best 2-D ultrasound images are obtained with a linear array probe (linear array intra-operative ultrasound, lioUS) (Philips, Amsterdam, The Netherlands), which is quite large and difficult to manoeuvre into a scalp window. For this reason, in a series of 13 LGG patients, Coburger et al. [23] compared a conventional phased array probe (conventional intra-operative ultrasound, cioUS) (Philips, Amsterdam, The Netherlands) with a new, smaller linear array probe. They observed that the lioUS images significantly corresponded to the intra-operative MRI (iMRI), and that it was unlikely that the cioUS was less accurate. The sensitivity was very high in the evaluation of the tumor residue for iMRI (83%), followed by lioUS (79%). The sensitivity for cioUS was lower (21%). On the other hand, in a series of 15 HGG patients, lioUS showed a significantly higher detection rate for residual tumors than cioUS, allowing a GTR of 73.3% [24]. Moreover, the authors found that the images produced by lioUS have few artefacts, better definition, and a more accurate visualization of the residual tumor compared with cioUS. Lothes et al. [25] in a prospective study on 11 patients with low-grade glioma (LGG) compared intraoperative MRI with conventional low-frequency intraoperative ultrasound (cioUS) and high-frequency linear array intraoperative ultrasound (lioUS). They concluded that although iMRI remains the method of choice, lioUS has been shown to be beneficial in a combined setup. Evaluation of lioUS was significantly superior to cioUS in most indications except for subcortical lesion. 

Proceeding further, the implementation of 3-D US should overcome some limitations of the 2-D US by producing a volumetric image. 3-D US showed 71% sensitivity in detecting residual tumors during the resection of cerebellar lesions, in a series of 16 patients who underwent resection of intracerebral lesions. However, the sample was too small to be conclusive [26]. Unsgaard et al. [27], in a study of 28 patients, analysed data of a 3-D IOUS-based intraoperative imaging and navigation system, comparing its usefulness in brain glioma and metastasis surgery. The results indicated that 3D US images give a good delineation of both metastases and the solid part of gliomas, thus providing a reliable guidance in tumor surgery before starting the resection. In larger series, it has been demonstrated that the use of 3-D navigable intraoperative ultrasound system may allow the surgeon to reach a 67% GTR of brain tumors [28].

Serra et al. [29], in a retrospective study of 22 patients, demonstrated that high frequency ultrasound (hfioUS) allows accurate detection of the tumor and detailed discrimination between normal, pathological, and oedematous tissue in all 22 cases, obtaining a GTR of 95.5%. Sweeney et al. [30], in a retrospective review of 260 patients, have shown that the use of IOUS might help to achieve a more successful GTR (81%) in both adult and paediatric neurosurgical patients. Moreover, a combination of IOUS with other intraoperative imaging modalities (such as fluorescent tissue enhancement) provided further increases of GTR in high-grade glioma surgery.

In our department, fluorescein sodium has been used as an adjunct in glioma resection since September 2015. We recently reported a resection >95% in 83% (*n* = 39) of patients who underwent fluorescence-guided surgery [31]. In recent years, our preliminary experience demonstrates that the combined use of fluorescence dyeing with B-mode ultrasonography and contrast-enhanced ultrasound (CEUS) techniques helps the surgeon recognize the boundary between normal brain parenchyma and tumor. In a technical note that is not yet published, we described the removal of high-grade gliomas under fluorescence dye, B-mode ultrasonoghraphy, and CEUS technique in five patients (3 males, 2 females; mean age 55.2 years, range 36–68 years) who underwent craniotomies for intra-axial lesions, which were suspected for high-grade gliomas on preoperative MRI. According to our experience, we confirm the utility of IOUS in the initial steps of surgery and the central role of fluorescence in achieving a GTR. Ultrasound-based neuronavigation provides intraoperative support in planning the craniotomy, localizing the lesion, choosing the best point for the corticectomy (especially if deep tumors), as well as for resection control checking the boundaries structures. On the other hand, fluorescence-guided surgery appears to be a surface phenomenon; it is very useful to identify and demarcate the tumoral tissue once it is sufficiently exposed; according to our opinion, fluorescein sodium appears to be more important in the latest steps of resection. In addition, we show the effectiveness, safety, accuracy, and feasibility of ultrasound-based fluorescein-guided surgery, which is less time- and cost-consuming. 

With regards to the prognosis of patients undergoing surgery for brain gliomas, it has been demonstrated that IOUS improves the prevalence of GTR and significantly increases 1- and 2-year overall survival [32]. These results may be due to the detection of residual tumors with high specificity by the use of IOUS, and hence, to the improvement of the resection rate [11]. In a series of 35 patients, Chacko et al. [33] reported that IOUS had a positive predictive value of 0.84, and Rygh et al. [34] showed similar results in a retrospective work of 19 high-grade glioma (HGG) (specificity and sensitivity of 95%). They reported a considerable decrease of specificity (up to 42%) during the resection, while the sensitivity remained as high as 87%. Nevertheless, after the resection, the sensitivity reaches a low value (26%), and the specificity has a value equal to 88%. Neuronavigation has undoubtedly provided great advantage in brain tumor surgery by improving surgical accuracy and safety. It is based on MRI or CT scans, which should be performed within 24 h prior to surgery. Unfortunately, after performing the craniotomy, changes in brain morphology may occur compared to preoperative examinations because of the brain shift [18], which can lead to inaccuracies of between 5 and 10 mm [35]. These changes also become even more important as the tumor is debulked. As some studies highlight, intraoperative ultrasound may allow us to overcome the limit of anatomic distortion due to brain shift and tumor debulking [36]. In this regard, in a series of 67 patients, Prada et al. demonstrated that brain shift distortion may be corrected by the fusion of images between intraoperative ultrasound and preoperative magnetic resonance using neuro-navigation systems [37,38]. They have concluded that intraoperative US imaging combined with neuro-navigator is reliable, accurate, and easy to use, allowing a continuous real-time feedback without interrupting surgery. 

With regards to tumor pathological characterisation, contrast-enhanced ultrasound (CEUS) is a valuable tool for visualizing vascularization patterns that often correlates with lesion histology. Prada et al. [39], in a series of 71 patients, found that intra-operative CEUS (iCEUS) allows for the characterization of different brain neoplasms. Furthermore, iCEUS shows afferent and efferent vessels and hyperperfused areas, thus possibly modifying the intraoperative surgical strategy. Arlt et al. [40], in a retrospective study of 50 patients, examined the advantages of using of contrast-enhanced and three-dimensional reconstructed ultra-sound (3D CEUS) in brain tumors. The authors found that three-dimensional CEUS is a useful intraoperative imaging tool, especially for brain glioma surgery. The results of our literature review are briefly summarized in Table 1.

## 4. Conclusions

The main objective in brain tumor surgery is to obtain a radical resection with minimal morbidity, as radical removal has been demonstrated to be a main factor affecting overall survival.

The advent of neuronavigation has certainly brought significant advantages in brain tumor surgery, allowing identification of the lesion and its margins during the resection, but there is the great limitation of anatomic distortion after craniotomy. Intraoperative ultrasound has allowed us to overcome this limit. Furthermore, IOUS exerts a notable attraction due to the low cost, minimal interruption of the operative flow, and lack of radiation exposure. In experienced hands, sonographic features can help differentiate low-grade gliomas, which can exhibit calcifications and mild hyperechogenicity from high-grade gliomas, which can show necrotic degeneration [61]. Our literature review shows that the increasing use of ultrasound in brain tumors may allow more radical resections, thereby increasing overall survival. The studies analysed in our review show a great correlation between postoperative MRI and intraoperative ultrasound, especially for gliomas and metastases. Moreover, the lioUS appears to provide higher quality images compared to the cioUS, particularly concerning the visualization of the tumor residual. Contrast-enhanced ultrasound (CEUS) allows for the evaluation of the tumor vasculature, thus suggesting the histological diagnosis. In conclusion, the combined use of IOUS and neuronavigation may facilitate tumor removal, enhancing more radical resection, and thus improving patient overall survival and quality of life.

## Figures and Tables

**Table 1:** Summary of the reviewed literature.

Author and Year	Study Design	N° Pts	Tumor Grade	GTR	Sen %	Sp%	Primary Endpoint	Results
Chacko et al., 2003 [33]	Clinical Article (Prospective Study)	35	HGG (22) LGG (11) Others (2)	12/35 (34.29%)	/	/	To evaluate the usefulness of intra-operative ultrasound (IOUS) in the detection of residual tumor compared with a postoperative computed tomogram and with histo-pathology.	The comparison between the IOUS findings and the post-op CT scan findings in the 28 pts with parenchymal tumors; 5 patients who had received prior radiation and 2 inflammatory granulomas were excluded from the analysis, there was concordance between the IOUS findings and the post-op CT scan in 23 of 28 cases.
Steno et al., 2012 [41]	Case Report (Retrospective)	1	LGG (1)	97%	/	/	/	/
Sæther et al., 2012 [21]	Retrospective study	192	HGG (192)	48/107 (45%) Vs. 34/45 (43%) operated before the introduction of intraoperative ultrasound	/	/	To examine if the introduction of 3D ultrasound and neuronavigation (i.e., the SonoWand® system) may have had an impact on overall survival.	Patient survival increased after introduction of intraoperative ultrasound and neuronavigation.
Erdogan et al., 2005 [22]	Original article (Prospective Study)	32	HHG 15 (GBM (8) Anaplastic astrocytoma (4) Oligodendroglioma (3)) Others (17)	59.38%	/	/	To determine the inter-method agreement between intraoperative ultrasono- graphy and postoperative contrast-enhanced magnetic resonance imaging (MRI) in detecting tumor residue.	Correlation with postoperative MRI revealed a good level of agreement (9 cases with agreement on positive residue and 19 cases with agreement on negative residue, no agree- ment in four cases)
Coburger et al., 2014 [24]	Prospective study	15	HHG 15 (GBM)	73.3%	/	/	To evaluate the use of navigated lioUS (linear array intraoperative ultrasound) as a resection control in glioblastoma surgery.	lioUS can be used as a safe and precise tool for intracranial image guided resection control of GBM. It shows a significant higher detection rate of residual tumor compared to conventional cioUS.
Moiyadi et al., 2013 [28]	Clinical Article (retrospective study)	90	HGG (51) LGG (17) Others (22)	67%			To assess the practical utility of 3D navigable US system and its impact on intraoperative decisions during cerebral glioma surgery and analyze the extent of resection achieved in malignant gliomas.	The navigable 3D US system is a very useful intraoperative image guidance tool in neuro-oncology, often facilitating better and radical resections.
PeredoHarvey et al., 2012 [42]	Original article (Prospective Study)	18	HHG 6 (GBM) LGG 3 (Oligodendroglioma) Others 9	85.6%	/	/	To test the feasibility of navigation based on ultrasound navigation only and analyze whether intraoperative imaging could substitute regular navigation for lesion localization for biopsies or resection and whether intraoperative imaging in this system allowed resection control.	Neuronavigation based solely on intraoperative ultrasound is feasible and may increase surgical safety when preoperative neuronavigational image is not feasible or unavailable.
Serra et al., 2012 [29]	Original article (Retrospective study)	22	HGG 14 Others 8	95.5%	/	/	To demonstrate the utility of intraoperative use of high frequency ultra- sound (hfioUS) in maximizing the extent of resection (EOR) of intracerebral high-grade tumors.	The hfioUS probe allowed in this study a precise detection of the tumor and a de- tailed discrimination between normal, patholog- ical and edematous tissue in all 22 cases.
Wang et al., 2012 [32]	Prospective Study	137	HGG (79) LGG (58)	81.8%	/	/	To investigate the value of intraoperative sonography in improving the prevalence of total tumor resection and the survival time of patients who underwent resection of cerebral gliomas.	The use of intraoperative ultrasound improves the prevalence of total tumor resection and the patient’s survival time.
Moiyadi et al., 2011 [20]	Original Article (retrospective analysis of prospectively collected data)	77 in 75 pts (one pts was operated three times) (69 brain tumors and 8 spinal timors)	41 glial tumors 36 others	76%	/	/	To evaluate the utility of the IOUS in an objective manner.	The IOUS is a very useful tool in intraoperative localization and delineation of lesions and planning various stages of tumor resection. It is easy, convenient, reliable, widely available, and above all a cost-effective tool.
Rohde et al., 2011 [26]	Prospective study	16	/	80.7%	71%	60%	To test if intraoperative 3-D ultrasound likewise can be used for resection control.	3-D ultrasound is especially helpful for detection of overseen brain tumor tissue.
Solheim et al., 2010 [43]	Clinical article (Retrospective Study)	142	HGG 142	74.5%	/	/	To evaluate resection grades and clinical outcome in surgery of high-grade gliomas, operated with use of the SonoWand system. To explore the impact of ultrasound image quality andrelationships between patient selection and surgical results.	The study suggest that better ultrasound facilitates better surgery and also clearly demonstrates that, in terms of surgical results, the selection of patients seems to be much more important than the selection of surgical tools.
Rygh et al., 2008 [34]	Clinical Article (Retrospective Study)	19	HGG 19	76.9%	95%	95%	To compare the ability of navigated 3D ultrasound to distinguish tumor and normal brain tissue at the tumor border zone in subsequent phases of resection.	This study shows that while ultrasound is highly accurate in delineating GBM before resection, but it appears less accurate during and after resection. During resection, there seems to be some overestimation of tumor, while small tumor remnants and infiltrated tissue in the cavity wall is underestimated after resection.
Lindner et al., 2006 [36]	Original Article (Prospective Study)	23	HGG 9 Others 14	77%	/	/	To prove the concept of 3D ultrasound with regard to technical effects and human impact. This includes measurement of fusion accuracy, the extent of tumor resection and the suitability for detection and capture of intraoperative brain shift as well as a protocol of operative handling as described by different neurosurgeons.	The introduction of 3D ultrasound has increased the value of neuronavigation substantially, making it possible to update several times during surgery and minimize the problem of brain shift.
Renner et al., 2005 [44]	Prospective Study	36	HGG 22 Others 14	76.2%	/	/	To evaluate intra-operative ultrasound (IOUS) as a tool of resection control after brain tumor surgery.	The reliability of IOUS depends on tumor type. It is beneficial to use IOUS for the resection of metastases and a few high-grade gliomas. Concerning the volumetric accuracy, the value of IOUS is worse than its value of navigation and resection control.
Unsgaard et al., 2005 [27]	Clinical Article (Prospective Study)	28	HGG 15 LGG 7 Others 6	76.6%	Low-grade astrocytoma: 72% Anaplastic astrocytoma: 86% Glioblastoma: 88% Metastasis: 100%	Low-grade astrocytoma: 100% Anaplastic astrocytoma: 75% Glioblastoma: 56% Metastasis: 100%	To investigate whether the images from the 3D US imaging system provide the surgeon with sufficient information to do a safe delineation of the margins of gliomas and metastases during the operation.	Reformatted images from 3D US volumes give a good delineation of metastases and the solid part of gliomas before starting the resection.
Mursc et al., 2017 [45]	Original Article (Prospective Study)	25	HGG 25	/	/	/	To investigate whether (IOUS) helped the surgeon navigate towards the tumor as seen in preoperative magnetic resonance imaging and whether IOUS was able to distinguish between tumor margins and the surrounding tissue.	During surgery performed on relapsed, irradiated, high-grade gliomas, IOUS provided a reliable method of navigating towards the core of the tumor. At the borders, it did not reliably distinguish between remnants or tumor-free tissue, but hypoechoic areas seldom contained tumor tissue.
Sweeney et al., 2018 [30]	Clinical Article (Retrospective review)	260	HGG 110 Others 150	81%	Glioma 50.8% Metastatic tumors 47.4%	Glioma 100% Metastatic tumors 100%	To expand on results from the previous study in order to provide more evidence on the usage of IOUS in the determination of gross-total resection (GTR) in both adult and pediatric patients with brain tumors.	The use of IOUS might help achieve a more successful GTR in both adult and pediatric neurosurgical patients and might improve surgical outcomes. It might be useful to study the combined efficacy of IOUS and intraoperative fluorescence imaging in achieving a higher GTR rate in invasive CNS tumor cases.
Sun et al, 2007 [46]	Original Article (Retrospective Study)	110	Gliomas 68 Others 42	/	/	/	To evaluate the value of IOUS in neurological operations.	IOUS was a valuable tool in localizing lesions, selecting the proper approach, con- trolling the extent of resection and displaying the distribution of vasculature. IOUS can provide more reliable safe guard for minimally invasive neurosurgery.
Smith et al., 2016 [47]	Clinical Article (Retrospective Review)	62	HGG 5 LGG 34 Others 23	71%	61.1%		To evaluate the correlation of extent of resection between IOUS and postoperative MRI.	The use of IOUS may play an important role in achieving a greater extent of resection by providing real-time information on tumor volume and location in the setting of brain shift throughout the course of an operation.
Lothes et al., 2016 [25]	Original Article (Prospective Study)	11	LGG 11	/	/	/	To evaluate the ideal application and typical interactions of intraoperative MRI (iMRI), conventional low-frequency intraoperative ultrasound (cioUS), and high-frequency linear array intraoperative ultrasound (lioUS) to optimize surgical workflow.	Although iMRI remains the imaging method of choice, lioUS has shown to be beneficial in a combined setup. Evaluation of lioUS was significantly superior to cioUS in most indications except for subcortical lesions.
Moiyadi et al., 2017 [48]	Original Article (Retrospective Study)	22	HGG 17 LGG 5	78%	/	/	To emphasize the convenience and feasibility of the use of navigable three-dimensional US with awake surgery for gliomas.	Combining awake surgery with 3DUS is feasible and beneficial. It does not entail any additional surgical workflow modification or patient discomfort. This combined modality intraoperative monitoring can be beneficial for eloquent region tumors.
Rueckriegel et al., 2016 [49]	Original Article (Retrospective Study)	11	/	27.27%	/	/	To assess whether the combined use of navigated ultrasonography with the integration of FMRIB Software Library based probabilistic fiber tracking into neuronavigations technically feasible and achievable in the preoperative and intraoperative workflow.	Integration of probabilistic fiber tracking and navigated ultrasonography into intraoperative neuro-navigation facilitated anatomic orientation during glioma resection. Combination with navigated ultrasonography provided a three-dimensional estimation of intra-operative brain shift and, therefore, improved the reliability of neuronavigation.
Pradaet al., 2016 [50]	Prospective study	10	HGG 10	/	/	/	To assess the capability of contrast-enhanced ultrasound (CEUS) to identify residual tumor mass during glioblastoma multiforme (GBM) surgery, to increase the extent of resection.	CEUS is extremely specific in the identification of residual tumor. The ability of CEUS to distinguish between tumor and artifacts or normal brain on B-mode is based on its capacity to show the vascularization degree. Therefore, CEUS can play a decisive role in the process of maximizing GBM resection.
Prada et al., 2015 [38]	Prospective Study	58	LGG 14 HGG 27 Others 17	/	/	/	To evaluate the role of intraoperative US imaging Associated whit conventional neuronavigation in brain tumor surgery.	Intraoperative US should be considered as a really valuable tool in guiding the surgeon’s hands in brain lesion removal, providing real-time feedback and allowing the operator to modify the surgical strategy based on the real intraoperative situation.
Prada et al., 2014 [39]	Prospective Study (in an off -label setting)	71	LGG 16 HGG 37 Others 18	/	/	/	To evaluate and describe different brain pathologies by means of intraoperative contrast-enhanced ultrasound (iCEUS) compared with preliminary baseline US and preoperative MRI. This technique, being dynamic and continuous, allows a real-time direct view of the vascularization and flow distribution patterns of different types of neurosurgical lesions.	iCEUS adds valuable anatomic and biological information such as vascularization, microcirculation, and tissue perfusion dynamic, which will possibly provide further insights into the pathology of brain tumors. It might help the surgeon to tailor the approach to the lesion, highlighting the lesion, clarifying between tumor and edematous brain tissue, and showing afferent and efferent vessels and hyperperfused areas, thus possibly modifying the intraoperative surgical strategy.
Prada et al., 2014 [51]	Prospective Study (in an off -label setting)	69	LGG 22 HGG 47	/	/	/	To perform the first characterization of cerebral glioma using CEUS and to possibly achieve an intraoperative differentiation of different gliomas.	CEUS is a fast, safe, dynamic, real-time, and economic tool that might be helpful during surgery in differentiating malignant and benign gliomas and refining surgical strategy.
Prada et al., 2014 [37]	Prospective study	67	/	/	/	/	To demonstrate the usefulness of US intraoperative use in conjunction with the navigation system as a guiding tool in brain tumor surgery.	Intraoperative US imaging combined with neuro-navigator represents a major innovation in neurosurgery; it is reliable, accurate, easy to use, permitting a continuous real-time feedback without interrupting surgery.
Policicchio et al., 2018 [52]	Original Artile (Retrospective Review)	162	HGG 62 LGG 9 Others 91	HGG 46.77% LGG 55.56% Metastases 86.67%	/	/	To assess the utility of routine use of iUS during various types of intracranial surgery.	US was highly sensitive in detecting all types of pathology, was safe and precise in planning trajectories to intraparenchymal lesions and was accurate in checking extent of resection in more than 80% of cases. iUS is a versatile and feasible tool; it could improve safety and its use may be considered in routine intracranial surgery.
Petridis et al., 2015 [53]	Retrospective Study	34 (15 pts ultrasound was used and in 19 not).	LGG 34	17.6%	/	/	To evaluate the importance of intraoperative diagnostic ultrasound for localization of low-grade gliomas.	Intraoperative ultrasound is an excellent tool in localizing low grade gliomas intraoperatively. It is an inexpensive, real time neuronavigational tool, which overcomes brain shift.
Neidert et al., 2016 [54]	Original article (Retrospective Study)	76	HGG 76	/	/	/	To analyze the impact of intraoperative resection control modalities on over- all survival (OS) and progression-free survival (PFS) following gross total resection (GTR) of glioblastoma.	OS and PFS were longer in patients that had a GTR using ioUS (either ioUS alone or ioUS in combination with ioMRI) compared to those patients without ioUS.
Moiyadi et al., 2016 [55]	Retrospective Study	111 (81 with US, 30without US)	HGG 75 LGG 12 Others 24	53%	/	/	To evaluate the effectiveness of Navigated 3D ultrasound as a novel intraoperative imaging adjunct permitting quick real-time updates to facilitate tumor resection	The results of this study demonstrate that 3D ultrasound can be effectively used as a stand-alone navigation modality during the resection of brain tumors. The ability to provide repeated, high-quality intraoperative updates is useful for guiding resection.
Lekht et al., 2016 [56]	Retrospective Study	5	HGG 1 LGG 1 Others 3	/	/	/	To provide further clinical data on the versatile application of Intraoperative contrast-enhanced ultrasound (iCEUS) through a technical note and illustrative case series.	iCEUS has potential for safe, real-time, dynamic contrast-based imaging for routine use in neurooncological surgery and image-guided biopsy. ICEUS eliminates the effect of anatomical distortions associated with standard neuronavigation and provides quantitative perfusion data in real time, which may hold major implications for intraoperative diagnosis, tissue differentiation, and quantification of extent of resection.
Ishikawa et al., 2017 [57]	Case Report (Retrospective Study)	15	HGG 5 LGG 2 Others 8	/	/	/	To evaluate the usefulness of the use of the latest innovative imaging technique for detecting very low-flow components, Superb Microvascular Imaging (SMI), with US during brain tumor surgery	US monitoring with SMI images in the gray scale mode is a pioneering monitoring technique to recognize tumor vessels and tumor margins and to differentiate tumor from surrounding healthy tissue.
Coburger et al., 2017 [58]	Prospective Study	33	HGG 33	/	80%	100%	To assess histopathological basis of imaging results of intraoperative magnetic resonance imaging (iMRI), 5-aminolevulinic acid (5-ALA), and linear array intraoperative ultrasound (lioUS).	All of the assessed established imaging techniques detect infiltrating tumor only to a certain extent. Only 5-ALA showed a significant correlation with histopathological findings.
Coburger et al., 2015 [23]	Clinical Article (Prospective Study)	13	LGG 13	/	79%	67%	To evaluate the accuracy of linear array ultrasound in comparison to conventional intraoperative ultrasound (cioUS) and intraoperative high-field MRI (iMRI).	Intraoperative resection control in LGGs using lioUS reaches a degree of accuracy close to iMRI. Test results of lioUS are superior to cioUS. cioUS often fails to discriminate solid tumors from “normal” brain tissue during resection control.
Coburger et al., 2015 [59]	Original Article (prospective non-randomized study)	20	HGG 20	/	76%	58%	To evaluate sensitivity and specificity of lioUS to detect residual tumor in patients harboring a glioblastoma.	Tumor detection using a lioUS is significantly superior to cioUS. Overall test performance in lioUS is comparable with results of iMRI, while, the latter has a higher specificity and a significantly lower sensitivity in comparison with lioUS.
Cheng et al., 2016 [60]	Clinical Study (Prospective Study)	88	HGG 50 LGG 38	/	/	/	To investigate the value of intraoperative contrast enhanced ultrasound (CEUS) for evaluating the grade of glioma and the correlation between microvessel density (MVD) and vascular endothelial growth factor (VEGF).	Intraoperative CEUS could help in determining boundary of peritumoral brain edema of glioma. Intraoperative CEUS parameters in cerebral gliomas could indirectly reflect the information of MVD and VEGF.
Arlt et al., 2016 [40]	Clinical Article (Prospective Study)	50	HGG 23 LGG 6 Others 21	GBM: 62%	/	/	To examine contrast-enhanced and three-dimensional reconstructed ultra- sound (3D CEUS) in brain tumor surgery regarding the up-take of contrast agent pre- and post-tumor resection, imaging quality and in comparison, with postoperative magnetic resonance imaging in different tumor entities.	Three-dimensional CEUS is a reliable intraoperative imaging modality and could improve imaging quality. Ninety percent of the high-grade gliomas (HGG, glioblastoma and astrocytoma grade III) showed high contrast uptake with an improved imaging quality in more than 50%. Gross total resection and incomplete resection of glioblastoma were adequately highlighted by 3D CEUS intraoperatively. The application of US contrast agent could be a helpful imaging tool, especially for resection control in glioblastoma surgery.
